# High Prevalence of Virulence-Associated Genes and Length Polymorphism in *actA* and *inlB* Genes Identified in *Listeria monocytogenes* Isolates from Meat Products and Meat-Processing Environments in Poland

**DOI:** 10.3390/pathogens13060444

**Published:** 2024-05-23

**Authors:** Iwona Kawacka, Agnieszka Olejnik-Schmidt

**Affiliations:** Department of Food Biotechnology and Microbiology, Poznan University of Life Sciences, Wojska Polskiego 48, 60-627 Poznan, Poland

**Keywords:** food safety, gene mutation, virulence, invasiveness, genotyping

## Abstract

*Listeria monocytogenes* is a human pathogen that has the ability to cause listeriosis, a disease with possible fatal outcomes. The typical route of infection is ingestion of the bacteria with contaminated food. In this study, 13 virulence-associated genes were examined with PCR in the genomes of 153 *L. monocytogenes* isolates collected from meat products and processing environments in Poland. All isolates possessed genes from LIPI-1—*hly*, *actA*, *plcA*, *plcB* and *mpl*—as well as four internalins: *inlA*, *inlB*, *inlC*, *inlJ*. Invasion-associated protein *iap*, as well as genes *prfA* and *sigB,* encoding regulatory proteins, were also detected in all isolates. Gene *flaA*, encoding flagellin, was detected in 113 (74%) isolates. This was the only gene that was not detected in all isolates, as its presence is serotype-dependent. Gene *actA* showed polymorphism with longer and shorter variants in PCR amplicons. Two isolates were characterized by truncated *inlB* genes, lacking 141 bp in their sequence, which was confirmed by gene sequencing. All isolates were positive in hemolysis assays, proving the synthesis of functional PrfA and Hly proteins. Four genotypes of *L. monocytogenes* based on *actA* polymorphism and two genotypes based on *inlB* polymorphism were distinguished within the isolates’ collection.

## 1. Introduction

*Listeria monocytogenes* is a Gram-positive, facultatively anaerobic rod adapted to various conditions. These ubiquitous bacteria are found throughout the environment. Many domestic animals, especially ruminants, are carriers of *L. monocytogenes*, which leads to the contamination of animal breeding areas, with the subsequent possibility of food contamination. The ingestion of *L. monocytogenes* with contaminated food may result in self-limiting gastroenteritis occurring with fever and diarrhea. Although usually listeriosis is mild, in severe cases symptoms include sepsis, meningitis, encephalitis, and spontaneous abortion. Overall mortality risk is estimated to be approximately 15% or higher, depending on patient status and comorbidities [1,2,3,4,5,6].

*L. monocytogenes* is an invasive intracellular pathogen. Its virulence depends on many adhesion and invasion factors that facilitate gastrointestinal tract colonization and crossing of the intestinal barrier [7]. Virulence factors also include proteins facilitating dissemination in the host, including brain and placenta colonization, cell-to-cell spread, the adhesion, and invasion of macrophages and escape from *L. monocytogenes*-containing vacuoles. Proteins enabling survival in the intestines, such as acid and bile tolerance proteins, are also virulence factors. The most important virulence-associated genes of *L. monocytogenes* are localized on Listeria pathogenicity island-1 (LIPI-1), namely, *hly*, *actA*, *plcA*, *plcB* and *mpl* [5,7,8,9]. All virulence-associated genes from LIPI-1 are positively regulated by a pleiotropic transcriptional regulator PrfA (encoded by the *prfA* gene), which is considered the main positive regulatory factor of virulence genes in *L. monocytogenes* [9,10,11].

Other groups of key listerial virulence factors, outside of LIPI-1, include internalins. The most important ones are *inlA* and *inlB*, but many have been identified (including *inlC*, *inlJ*, *inlH*, *inlK*, *inlL*, *inlF* and *inlP*) [8,9,12,13]. Other known listerial virulence factors include, for example, invasion-associated protein (encoded by *iap*), flagellin (encoded by *flaA*), a general stress-response regulator, called sigma factor B, (encoded by *sigB*), Listeria-mucin-binding invasin A, bile salt hydrolase, and cell invasion LPXTG protein, ClpP, a heat shock protein that is involved in intracellular growth or fibronectin-binding protein [7,8,10,12], to just name a few. However, not all *L. monocytogenes* isolates harbor all discovered virulence genes [14,15,16].

A species-specific characteristic of *L. monocytogenes*, historically considered to be a virulence marker, is beta-hemolytic activity [17]. This trait is often used to confirm the species identification of the isolates [4,18,19]. However, there are also reports about isolates of this species that do not present hemolytic phenotype, mainly due to mutations within either the *hly* gene, or, more frequently, *prfA* mutations [20]. Furthermore, it has been suggested that the spontaneous loss of virulence in natural populations of *L. monocytogenes*, although rare, is possible due to the fact that some of the virulence genes are under purifying selection. This opens an evolutionary path for potential saprophytism for this pathogen [20]. Hence, tracking potential changes in the virulence-associated genes patterns in food isolates of *L. monocytogenes* is important, as a trend of reducing pathogenicity in this genus may be observed.

## 2. Aim of This Study

The aim of this study was to assess the diversity of *L. monocytogenes* isolates collected in recent years in Poland originating from meat products and meat processing facilities. The presence or absence (or polymorphic form) of selected virulence-associated genes was assessed and the hemolytic phenotype of those isolates was determined. An estimation of the virulence potential of the collected isolates based on the obtained results was an additional aim of this study.

## 3. Materials and Methods

### 3.1. Bacterial Isolates and Genetic Material

A collection of 153 *L. monocytogenes* isolates used in this study originated from both raw meat samples and processed meat products manufactured in Poland (*n* = 108), as well as from meat processing plants in Poland (*n* = 45), representing food processing and environmental surfaces. The isolates were collected between October 2020 and November 2021. The DNA of those microorganisms used for gene detection was isolated using a Genomic Mini kit (A&A Biotechnology, Gdańsk, Poland) according to the manufacturer’s instructions. Bacterial isolates preserved in brain heart infusion broth (BHI; Oxoid, Warsaw, Poland) glycerol stocks stored at −80 °C were used for hemolysis assays. Isolates included in the study were confirmed as *L. monocytogenes* species with two separate genetic analyses, using a PCR-RFLP according to Paillard et al. (2003) [21] and multiplex PCR according to Li et al. (2021) [22] protocols. Details regarding the isolates’ collection process, DNA isolation procedure, and the exact methodology for species affiliation were reported previously [23].

### 3.2. Detection of Virulence-Associated Genes

The presence of thirteen virulence-associated genes in genomes of *L. monocytogenes* isolates was analyzed in the study, using standard PCR for most of the genes or multiplex PCR for the simultaneous detection of the two genes *inlB* and *inlC*. If an amplicon for any gene was not detected in a multiplex reaction, then separate PCRs using only one pair of primers were performed for verification. If discrepancies occurred between multiplex and singleplex, only the singleplex results were taken into account. Additionally, two pairs of primers (here referred to as *actA1* and *actA2*) were used to analyze the presence of the *actA* gene.

Reaction mixtures contained 0.2U of RUN polymerase (A&A Biotechnology), along with compatible reaction buffer at the recommended concentration, 0.2 mM of each dNTPs (A&A Biotechnology) and primers (concentrations are given in Table 1), which were ordered in Genomed S.A. (Warsaw, Poland). DNA was added in the amount of 10 ng per reaction. Reactions were performed in a final volume of 10 μL. Thermal cycling was performed in the T-Gradient thermocycler (Biometra, Göttingen, Germany) with annealing temperatures, as presented in Table 1. PCR products were separated by electrophoresis in an agarose gel-containing ethidium bromide and visualized using Gel Doc Imaging System (Bio-Rad, Hercules, CA, USA).

At least one randomly chosen sample representing one amplicon size amplified with one primer pair was sequenced. PCR products were purified prior to sequencing directly from the PCR mixture or after separation in an agarose gel, using the A&A Biotechnology kits, with either Clean-Up Concentrator or Gel-Out Concentrator. Sequencing was performed by Genomed S.A. Obtained sequences were determined to be fragments of genes of interest using Blast software (BLASTN 2.14.1+) [35,36].

In the case of *inlB* gene, in order to obtain a full-length sequence for more reliable reads, sequences achieved with the forward primer were aligned with the sequence achieved using the reverse primer. BLAST [37] was used for this purpose. The obtained aligned sequences (for isolates 50 and 235) were compared with each other as well as with the *inlB* gene sequence of *L. monocytogenes* EDG-e from the GenBank database (accession number NC_003210.1), also using BLAST.

### 3.3. Hemolysis Assay

Hemolysis assays were performed on a commercially available Columbia Agar with 5% Sheep Blood (Becton, Dickinson and Company, Heidelberg, Germany). Bacterial isolates from glycerol stocks were streaked into BHI agar plates and incubated for 18 h at 37 °C. After incubation, bacteria were re-streaked from BHI to the blood agar plate and incubated at 37 °C for 24 h and 48 h, with assessment after both time periods. *Listeria ivanovii* ATCC 19119 was used as a positive control and *Listeria innocua* food isolates 135, 145, and 159 were used as negative controls.

## 4. Results

### 4.1. Presence of Virulence-Associated Genes

In the case of ten tested virulence-associated genes, namely, *iap*, *sigB*, *prfA*, *hlyA*, *inlC*, *inlA*, *inlJ*, *plcA*, *plcB*, and *mpl,* all of the *L. monocytogenes* isolates included in the study were positive in PCR with amplicons of expected length, in accordance with the literature references. The results were further confirmed with sequencing and BLAST analyses. An amplicon of the *flaA* gene was present in 113 (74%) isolates, all of which belonged to genoserotype IIa (representing serotypes 1/2a, 3a) (results reported previously; see Reference [23]). Furthermore, all isolates representing genoserotype IIa harbored the *flaA* gene [23]. 

In the case of actA and inlB genes, polymorphism of amplicon sizes was detected within the isolates’ collection. Summarized virulence detection results are presented in Table 2.

In the case of /*actA*/ and /*inlB*/ genes, polymorphism of amplicon sizes was detected within the isolates’ collection. Summarized virulence detection results are presented in Table 2 above.

### 4.2. actA Polymorphism

Typing *L. monocytogenes* using the two *actA* primer pairs allowed for four genotypes to be differentiated, which are presented in Table 3.

Interestingly, there is no clear pattern between genoserotype, as determined using the protocol of Doumith et al. protocol [38] (results published previously; see Reference [23]), and the *actA* amplicon variants achieved in this study, which allowed for the further differentiation of genoserotyped isolates.

**Figure 1:**
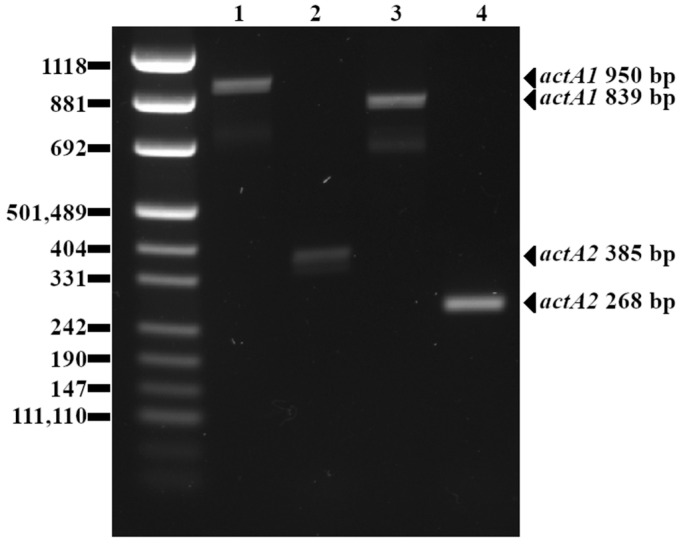
Agarose gel (2%) electrophoresis of DNA fragments generated with PCR using both *actA1* and *actA2* primers. Lanes 1 and 2: *L. monocytogenes* 234 (longer PCR amplicon achieved with *actA1* and *actA2*, respectively); Lanes 3 and 4: *L. monocytogenes* 45 (shorter PCR amplicon achieved with *actA1* and *actA2*, respectively).

Polymorphic variants of the *actA* gene detected with *actA1* and *actA2* primer pairs are presented in Figure 1 below.

### 4.3. inlB Polymorphism

Two isolates presented a shorter *inlB* amplicon than expected. Both of them are characterized by shorter variants of the *actA* gene, both belonged to the same rare genoserotype IIb (representing serotypes 1/2b, 3b, 7), and both shared the same antibiotic resistance profile, with susceptibility to all 10 tested antibiotics (results published previously; see Reference [39]), although the vast majority (95%) of isolates from this study were characterized by this particular susceptibility profile.

The two isolates with the truncated *inlB* gene originated from head cheese (processed meat product, also known as brawn) (isolate 50) and the Vienna-type sausage (isolate 235). Sequences of the *inlB* genes of those two isolates, when aligned with the BLAST, showed 357/366 (99%) identities. When compared to the EGD-e strain, both isolates 50 and 235 lack the 141-bp long fragment (encoding the β-repeat sheet and partially encoding the GW1 domain of the InlB protein [40]) within the *inlB* gene sequence.

**Figure 2:**
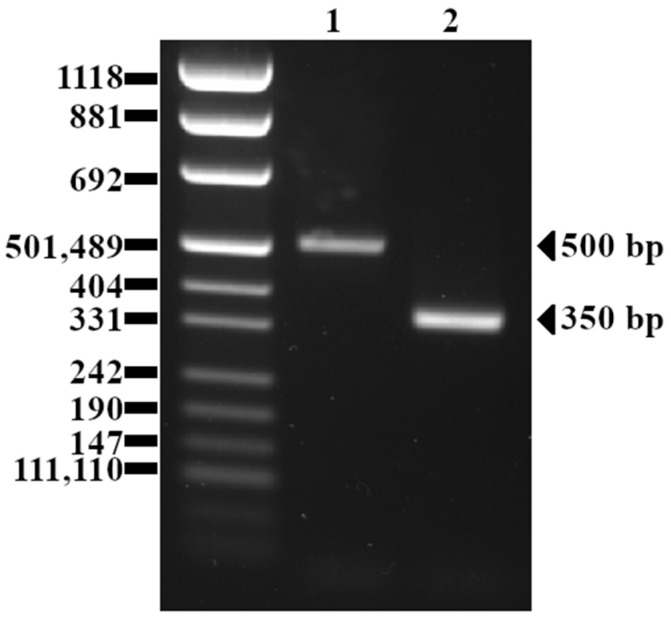
Agarose gel (2%) electrophoresis of DNA fragments generated with PCR using *inlB* primers. Lane 1: *L. monocytogenes* 99B (full-length PCR amplicon); Lane 2: *L. monocytogenes* 235 (with a deletion in *inlB* gene).

The detected variants of the *inlB* gene are presented in Figure 2 below.

### 4.4. Hemolysis

All 153 tested *L. monocytogenes* isolates presented the hemolytic phenotype. The response was consistent after 24 h and 48 h incubation. Positive results for the hemolysis assay indicate that Hly (listeriolysin O) encoded by the *hly* gene is functional, and that the PrfA regulator, encoded by *prfA*, positively regulating *hly* expression, also remains active.

## 5. Discussion

### 5.1. General Prevalence of Virulence-Associated Genes

Recent studies of *L. monocytogenes* originating from Poland do not always confirm the prevalence of all tested virulence-associated genes in bacterial genomes [14,15]. For example *hlyA* and *prfA* were present in 100% of the 27 food isolates and 13 isolates from the food processing environments, whereas *inlB* and *sigB* genes were present in 26 (97.5%) and 20 (82.5%) of the samples, respectively [14]. Genes *plcB*, *hlyA*, *iap*, *actA*, *prfA*, and *sigB* were present in all seven isolates originating from fish housed in Poland, whereas *inlB* was detected in six (85.7%) isolates [15]. However, similarly to our results, internalin family member genes *inlA*, *inlB*, *inlC*, *inlE*, *inlF*, and *inlJ* and the pathogenicity island LIPI-1 were found in 48 (100%) of the tested isolates from different kinds of ready-to-eat (RTE) food of animal origin and from a food processing environment in Poland [40]. Interestingly, the authors also identified one isolate with a deletion of 141 nucleotides in the *inlB* gene [40].

In recent international studies, some authors report the presence of the tested virulence genes in 100% of the examined isolates (including, e.g., *hlyA*, *prfA*, *iap*, *inlA*, *inlB*, *mpl*, *plcA*, and *plcB* [41]), whereas sometimes particular genes were detected only in a subgroup of samples, e.g., only 70% and 80% of the isolates from bovine farms in India harbored *plcA* and *plcB* genes, even though *hlyA* and *iap* were present in all of them [16].

The only gene not detected in all isolates in our study is *flaA*, encoding flagellin, which is a protein specific to the 1/2a and 3a serotypes [34]. All isolates positive for the gene encoding flagellin (74%) belong to genoserotype IIa (representing serotypes 1/2a, 3a) [23], and all isolates that were negative belong to other genoserotypes, which is consistent with the theoretical expected results.

### 5.2. actA Polymorphism

A polymorphism within the *actA* gene of *L. monocytogenes* isolates has already been reported in the literature [42,43]. Furthermore, the partial sequencing of this gene has been used as a tool for subtyping *L. monoctogenes* isolates, enabling the division of the isolates into two [42] or three [43] lineages. In our study, we identified four genotypes based on *actA* typing using two sets of primers.

Interestingly, the *actA* gene is located on LIPI-1 between the *mpl* and *plcB* genes [44] and, in our study, both flanking genes were identified in all 153 isolates, without detecting any visible polymorphism. This raises questions about the origin of variability within this particular gene, especially considering that the genotypes established based on *actA* are not correlated with serotypes, which was proved by our study and also reported earlier by other researchers [45].

Although there are studies indicating that the in vitro virulence of *L. monocytogenes* is not determined by the *actA* polymorphism [46], some publications found that the *actA* polymorphism influences the virulence potential of the isolates [42,43,45]. In one study, isolates classified based on the *actA* polymorphism as lineage II showed significantly lower invasiveness on epithelial Caco-2 cells than lineage I isolates [43]. Similarly, in the paper where *L. monocytogenes* was subtyped with this method, isolates from lineage I all contained highly invasive isolates, as well as isolates with moderate and low invasiveness, whereas lineage II contained only low-invasive isolates. The invasiveness was established with a cell-invasion assay with the CX-1 human colon cancer cell line [42]. Earlier studies indicated that the deletion of one large unit within a proline-rich region of ActA resulted in a reduction in intracellular bacterial speed, as well as decreased virulence [45]. The primer pairs (*actA1* and *actA2*) used herein include regions translated to proline-rich regions of the protein.

### 5.3. inlB Polymorphism

Two isolates from the collection examined in this study presented a truncated *inlB* gene with a 141 bp deletion. Interestingly, Kurpas et al. (2020) also identified an isolate from Poland with 141 bp deletion in the same region of *inlB* gene [40].

There are also other reports about *L. monocytogenes* strains harboring mutations within this gene. For example, a food isolate from Mexican-style soft cheese *L. monocytogenes* F2365 harboring mutations resulting in premature stop codons in *inlB* was characterized by a reduced invasion efficiency in Caco-2 cells [47]. The same isolate with introduced point mutation resulting in rescued *inlB* expression showed approximately 9-fold and 1.5-fold higher invasion in HeLa and JEG-3 cells, respectively, when compared to the parental strain [48]. These findings are consistent with a study in which a constructed *inlB* deletion *L. monocytogenes* mutant showed significantly decreased invasiveness in a mouse as compared to wild-type isolate [49]. However, there is also a report indicating that the *L. monocytogenes* A23 strain with inactive internalin B protein remained virulent in a plaque assay on human adenocarcinoma cell line HT-29 [50]. Interestingly, one study demonstrated that InlB domain variants may differ in their ability to support intragastric infection (measured as bacterial loads in livers of intragastrically infected mice), even though, in a cell culture study (measuring the invasion rate of murine colon carcinoma C26 cells), the results were not clearly apparent [51].

### 5.4. Hemolysis

Hemolysis assay is a phenotypic criterion used for confirmation of the species affiliation of collected isolates [29,52,53,54]. However, there are reports stating that some *L. monocytogenes* isolates do not present with hemolysis, with a rate of approximately 0.1% according to a study which included 57,820 isolates of food, clinical, veterinary, environmental, and other origins [20]. A lack of hemolytic phenotype can indicate diminished virulence caused by either a nonfunctional Hly protein due to mutations or its hampered expression due to mutations in the *prfA* gene, encoding the PrfA regulator that positively regulates Hly expression [20].

Hemolysis assays in the case of *L. monocytogenes* may provide interpretative difficulties. There is no consistency within the literature regarding the most sensitive method for hemolysis assessment or the blood type that would provide optimal test sensitivity [55]. Similarly to many other authors [20,56,57,58,59,60,61,62,63], we decided to apply the blood agar technique. Due to the fact that we observed weaker hemolysis in the case of *L. monocytogenes* strains than in the positive control, we do not recommend using *L. ivanovii* ATCC 19119 for that purpose, as this factor could contribute to false-negative reads in the case of *L. monocytogenes* isolates.

## 6. Summary and Conclusions

Although all 153 isolates possessed the 12 tested genes (*iap*, *sigB*, *prfA*, *hly*, *actA*, *inlB*, *inlC*, *inlA*, *inlJ*, *plcA*, *plcB*, and *mpl*), a PCR detection of virulence-associated genes allowed us to differentiate *L. monocytogenes* strains in our collection. The presence of the *flaA* gene was strictly serotype-dependent, whereas a polymorphism found in the *actA* gene could further differentiate strains pre-grouped into serogroups. Four major genotypes were identified based on *actA* typing. Two isolates were also differentiated from the rest of the collection due to a 141 bp deletion within the *inlB* gene sequence.

In terms of the prevalence of virulence-associated genes, our results are in agreement with the literature. Some authors report an absence of particular genes (e.g., *plcA*, *plcB*, *sigB*, *inlB*) in a subset of samples, whereas all of them were detected in all isolates included in our study. However, we did not observe that our results broke with any clear trend presented in the literature. The prevalence of particular genes in *L. monocytogenes* isolates depends on the strain collection itself and minor discrepancies are not only unsurprising but even expected. Due to this fact, the research on this topic is ongoing.

Based on the results and the available literature, we cannot draw conclusions regarding the potentially diminished virulence of some isolates in our isolates collection. However, isolates may differ in terms of their invasiveness due to the presence or absence of other virulence-associated genes that are not included in our study. Furthermore, gene mutations, even those that are undetectable with PCR, may lead to the diminished activity of a synthesized protein or the premature stopping of synthesis. In our study, we detected polymorphic forms in the case of *actA* and *inlB* genes, which may influence virulence potential. However, there is no literature consensus about the impact of those two genes on the invasiveness. The hemolytic phenotype observed in all isolates was confirmed to have hemolytic activity, proving that the Hly protein, as well as its regulator PrfA, are functional.

This study provided up-to-date knowledge about the high rate of prevalence of virulence-associated genes in the genomes of *L. monocytogenes* included in the study. Further research about the importance of detected *inlB* mutations in terms of the invasion rate should be performed in order to establish its influence on the virulence potential of isolates carrying this mutation.

## Figures and Tables

**Table 1:** Sequences of primers used in the study and cycling condition information.

Primers Name	Target	Primers’ Sequence	Primer Concentration [µM]	Annealing Temperature [°C]	Amplicon Length (bp)	Reference
*prfA*	Listeriolysin positive regulatory protein	F: 5′-GATACAGAAACATCGGTTGGC-3′ R: 5′-GTGTAATCTTGATGCCATCAGG-3′	0.3	49	274	[24]
*sigB*	Sigma factor	F: 5′-TCATCGGTGTCACGGAAGAA-3′ R: 5′-TGACGTTGGATTCTAGACAC-3′	0.35	51	310	[25]
*plcA*	Phosphatidylinositol-specific phospholipase C	F: 5′-CTGCTTGAGCGTTCATGTCTCATCCCCC-3′ R: 5′-CATGGGTTTCACTCTCCTTCTAC-3′	0.5	60	1484	[26]
*plcB*	Phosphatidylicholin-specific phospholipase C	F: 5′-GCAAGTGTTCTAGTCTTTCCGG-3′ R: 5′- ACCTGCCAAAGTTTGCTGTGA-3′	0.5	55	795	[27]
*hly*	Listeriolysin O	F: 5′-GCAGTTGCAAGCGCTTGGAGTGAA-3′ R: 5′-GCAACGTATCCTCCAGAGTGATCG-3′	0.3	62	456	[28]
*actA1*	Actin polymerization protein	F: 5′- CGCCGCGGAAATTAAAAAAAGA-3′ R: 5′- ACGAAGGAACCGGGCTGCTAG-3′	0.4	62	839 (or 950)	[29]
*actA2*	Actin polymerization protein	F: 5′-GACGAAAATCCCGAAGTGAA-3′ R: 5′-CTAGCGAAGGTGCTGTTTCC-3′	1.0	63	268 (or 385)	[30]
*mpl*	Metalloprotease	F: 5′-GGCTCATTTCACTATGACGG-3′ R: 5′- GCTTCCCAAGCTTCAGCAACT-3′	0.5	60	143	[27]
*inlA*	Internalin A	F: 5′-ACGAGTAACGGGACAAATGC-3′ R: 5′-CCCGACAGTGGTGCTAGATT-3′	0.5	55	800	[31]
*inlB*	Internalin B	F: 5′- CATGGGAGAGTAACCCAACC-3′ R: 5′- GCGGTAACCCCTTTGTCATA-3′	0.75	57	500	[32]
*inlC*	Internalin C	F: 5′- CCCACAATCAAATAAGTGACCTT-3′ R: 5′- CTGGGTCTTTGACAGTATTTGTT-3′	1.25	57	400	[32]
*inlJ*	Internalin J	F: 5′-TGTAACCCCGCTTACACAGTT-3′ R: 5′-AGCGGCTTGGCAGTCTAATA-3′	0.5	55	238	[31]
*iap*	Invasion associated protein	F: 5′-ACAAGCTGCACCTGTTGCAG-3′ R: 5′-TGACAGCGTGTGTAGTAGCA-3′	0.3	56	131	[33] *
*flaA*	Flagellin	F: 5′-TTACTAGATCAAACTGCTCC-3′ R: 5′-AAGAAAAGCCCCTCGTCC-3′	1.0	54	538	[34]

* referred to as presumptive β-hemolysin gene in Reference [33].

**Table 2:** Summarized results of virulence genes’ detection.

Primer Name	Approx. Amplicon Size (bp)	Number of Isolates (%)
*prfA*	274	153 (100%)
*sigB*	310	153 (100%)
*plcA*	1484	153 (100%)
*plcB*	795	153 (100%)
*hlyA*	456	153 (100%)
*actA1*	839	15 (10%)
	950	120 (78%)
	no amplicon	18 (12%)
*actA2*	268	28 (18%)
	385	125 (82%)
*mpl*	143	153 (100%)
*inlA*	800	153 (100%)
*inlB*	500	151 (99%)
	360	2 (1%)
*inlC*	400	153 (100%)
*inlJ*	238	153 (100%)
*iap*	131	153 (100%)
*flaA*	538	113 (74%)
	no amplicon	40 (26%)

**Table 3:** Number of isolates presenting the amplicon variants with particular *actA* primer pairs.

	*actA1* Shorter Amplicon (839 bp)	*actA1* Longer Amplicon (950 bp)	*actA1* No Amplicon
*actA2* shorter amplicon (268 bp)	15	0	13
*actA2* longer amplicon (385 bp)	0	120	5

## Data Availability

The data presented in this study are available on request from the corresponding authors.
